# The complete mitochondrial genome of *Eulaelaps huzhuensis* (Mesostigmata: Haemogamasidae)

**DOI:** 10.1007/s10493-023-00802-6

**Published:** 2023-06-22

**Authors:** Hui-Juan Yang, Zhi-Hua Yang, Tian-Guang Ren, Wen-Ge Dong

**Affiliations:** 1 Institute of Pathogens and Vectors, Yunnan Provincial Key Laboratory for Zoonosis Control and Prevention, Dali University, Dali, 671000 China; 2 School of Foreign Languages, Dali University, Dali, 671000 China; 3 College of Nursing, Dali University, Dali, 671000 China

**Keywords:** Haemogamasidae, Phylogenetic analysis, Mitochondrial genome, Gene rearrangement

## Abstract

Some mites of the family Haemogamasidae can transmit a variety of zoonotic diseases and have important public health and safety implications. Currently, however, little attention has been paid to molecular data of Haemogamasidae species, limiting our understanding of their evolutionary and phylogenetic relationships. In this study, the complete mitochondrial genome of *Eulaelaps huzhuensis* was determined for the first time, and its genomic information was analyzed in detail. The mitochondrial genome of *E. huzhuensis* is 14,872 bp in length with 37 genes and two control regions. The base composition showed a distinct AT preference. Twelve protein-coding genes have a typical ATN as the start codon, and three protein-coding genes have incomplete stop codons. During the folding of tRNA genes, a total of 30 mismatches occurred, and three tRNA genes had an atypical cloverleaf secondary structure. The order of the *E. huzhuensis* mitochondrial genome arrangement is a new type of rearrangement in Mesostigmata. The phylogenetic analysis confirmed that the family Haemogamasidae is a monophyletic branch and does not belong to a subfamily of the Laelapidae. Our results lay the foundation for subsequent studies on the phylogeny and evolutionary history of the family Haemogamasidae.

**Supplementary Information:**

The online version contains supplementary material available at 10.1007/s10493-023-00802-6.

## Introduction

Mites of the family Haemogamasidae (Mesostigmata, Dermanyssoidea) are widely distributed, with 78 species in five genera currently recorded (Beaulieu et al. 2011; Vinarski and Korallo-Vinarskaya 2017). Haemogamasidae mites are closely related to medicine; Baker and Wharton (1952) reported that they can transmit various zoonotic diseases, such as plague, typhoid fever of the intestine, tularemia, and some other diseases.

The genus *Eulaelaps* is a relatively small genus with mites commonly found in damp places, straw, and soil (Uchikawa and Rack 1979). Some mites of the genus *Eulaelaps* are ectoparasitic on small mammalian hosts and can be found on the body surface of rodent species (Netušil et al. 2013). As bloodsuckers and predators, they are widely distributed throughout the world (Uchikawa and Rack 1979). Turk (1945) and Allred (1969) considered *Eulaelaps stabularis* to be the most common mite found on the nests and bodies of small rodents and insectivores. Some *Eulaelaps* mites are considered to be important vectors of zoonotic diseases, such as *E. stabularis* and *Eulaelaps shanghaiensis*, which can transmit hemorrhagic fever with renal syndrome virus (HFRS) (Huang et al. 2013). Interestingly, *E. stabularis* is not only parasitic on rodents, but is also commonly found in agricultural silos, feeding on grains, other mites, and insect excreta (Makarova 2013). Ren et al. (2009) found *Eulaelaps huzhuensis* had minimal host specificity.

The genus *Eulaelaps* was originally described as a subgenus before it was elevated to a separate genus. The taxonomic status of the genus *Eulaelaps* has been controversial, with Oudemans (1913), Baker and Wharton (1952), and Bregetova (1956a) classifying the genus *Eulaelaps* as the family Laelapidae. However, in the late 1950s, Strandtmann and Wharton (1958) classified the genus *Eulaelaps* into the family Haemogamasidae, considering that the morphological characters in *Eulaelaps* species were similar to those of mites in the family Haemogamasidae. Later, Domrow (1987), Deng et al. (1993) and Mašán and Fenďa (2010) downgraded the family Haemogamasidae to the subfamily Haemogamasinae and added the subfamilies Laelapinae, Hypoaspidinae, and other subfamilies to the family Laelapidae. However, Haitlinger (1988), Goncharova et al. (1991), Vinarski and Korallo-Vinarskaya (2017) still consider the family Haemogamasidae as a separate family, and this taxonomic status is supported by Dowling and OConnor (2010) using molecular phylogenetic methods.

In recent years, with the development of molecular biology, especially the wide application of PCR and sequence determination techniques, more and more mitochondrial genomes (mitogenomes) of arthropods have been sequenced, gradually deepening our understanding of arthropod DNA aspects. Mitochondrial genomes have been widely used in species identification, genetic evolution, phylogeny, genealogical geography, and population genetics because of their simple structure, conserved gene content, matrilineal inheritance, high copy number, rapid evolutionary rate, and rare recombination (Tatarenkov and Avise 2007; Yang et al. 2022a, b). Mesostigmata is the most species-rich group of Parasitiformes, with approximately 11,424 species worldwide (Bolger et al. 2018). To date (Januray 2023), the mitochondrial genomes of only 23 species in Mesostigmata have been sequenced (including only the sequences of annotated genes) (Navajas et al. 2002; Jeyaprakash and Hoy 2007; Swafford and Bond 2009; Dermauw et al. 2010; Xin et al. 2014; Li et al. 2019; Wu et al. 2019; Osuna-Mascaró et al. 2020; Zhang et al. 2021; Ma et al. 2022; Yang et al. 2022a), and the NCBI database contains more ‘barcode’ sequences such as *cox1*, *12S rRNA*, *16S rRNA*, etc. The scarcity of sequence data has greatly hindered our study of phylogenetic relationships among Mesostigmata species. Hence, the complete mitochondrial genome may help increase our understanding of the evolutionary history of different taxa and their phylogenetic relationships.

Given that some mites of the genus *Eulaelaps* are capable of transmitting zoonotic diseases, it can have an important impact on public health safety. However, most studies on the genus *Eulaelaps* have focused on the description of morphological characters or the study of the pathogens they carry, whereas molecular data have been neglected, so that no mitochondrial genome of the mites in the family Haemogamasidae has been reported. In this study, the mitochondrial genome of *E. huzhuensis* was sequenced for the first time, and further phylogenetic analysis was performed using 13 protein-coding genes during the analysis of its mitochondrial genomic characteristics. The results not only fill a gap in molecular data information for the Haemogamasidae but also provide a scientific reference for the subsequent study of population genetic variation, molecular classification and identification, and the phylogeny of mites in the family Haemogamasidae.

## Materials and methods

### Mite collection

*Apodemus chevrieri* was captured with mousetraps, and 28 mites were collected from the body surface in Hongyuan County, Aba Tibetan and Qiang Autonomous Prefecture, Sichuan Province, China, in October 2021. The collected mite specimens were stored in EP tubes with 95% ethanol and numbered. The small mammal capture protocols and procedures followed were approved by the animal ethics committees at Dali University. The approval ID is MECDU-201806-11.

### Morphological identification, DNA extraction and mitogenome sequencing

**Fig. 1:**
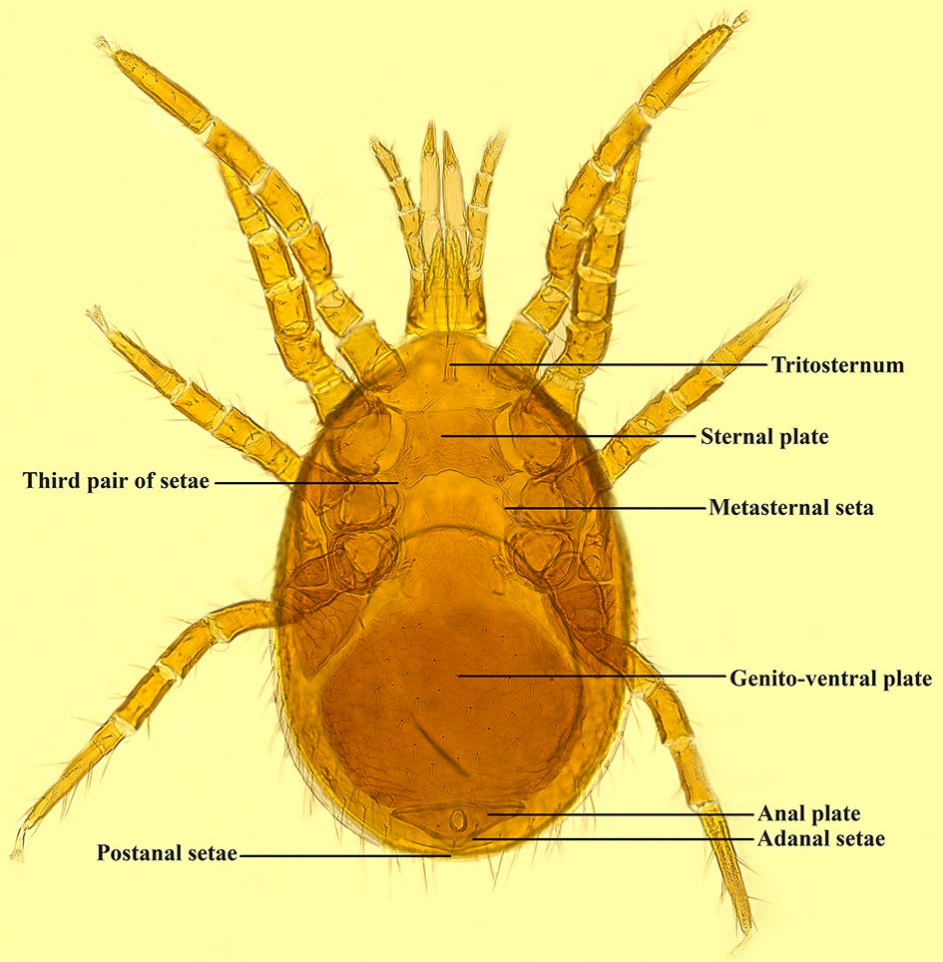
Morphological characters of *Eulaelaps huzhuensis*

The 28 mites were removed from the EP tubes containing 95% ethanol and morphological identification of the mites was performed under the SZ2-ILST dissecting microscope (Olympus, Tokyo, Japan) based on Deng et al. (1993). The mites were identified as *E. huzhuensis*. *Eulaelaps huzhuensis* is mainly identified by the following characteristics: anterior region of the sternal plate with 5–6 reticulations, tritosternum tip smaller. Sternal plate with three pairs of setae and two pairs of lyriform fissure, and densely reticulated; posterior margin depressed deeper, base of depression exceeding the level for the third pair of setae. Metasternal seta inserted on epidermis, medially with a lyriform fissure. Genito-ventral plate lateral margin notched at genital plate and ventral plate fusion, but not obviously concave, with 55 (49–58) setae on ventral plate area. Anal plate broadly triangular, adanal setae located on anus mid-transverse line, approximately equal to postanal setae (Fig. 1).

The mites were immersed in sterile distilled water for 30 min to remove microorganisms from the surface. Genomic DNA was extracted using the DNeasy Blood and Tissue Kit (Qiagen). Short fragments of *cox1* genes were initially amplified by polymerase chain reaction (PCR) using primer pairs cox1F (CATTTTCHACTAAYCATAARGATATTGG) and cox1R (TATAAACYTCDGGATGNCCAAAAAA) (Dabert et al. 2010). The obtained amplification products were sequenced directly at Majorbio (Shanghai, China) using the Sanger method to obtain partial sequences of the *cox1* gene. Then the specific primers mcox1F (GTTGAAAGAGGGGCTGGAACAGGTTGAAC) and mcox1R (GTCTGGGGCAGAAATTATTATTGGGACC) of *E. huzhuensis* were further designed based on the obtained short fragment of the *cox1* gene, and PCR with the specific primers generated an amplicon fragment of about 15 kb. Takara EX *Taq* (Takara Biomedical, Japan) was used for short fragment amplification, and the PCR reaction conditions were as follows: 94 ℃ pre-denaturation for 2 min; 94 ℃ denaturation for 30 s; 56–60 ℃ annealing for 30 s; 54–72 ℃ extension for 1 min, 35 cycles, and 72 ℃ extension for 10 min. The Takara LA *Taq* was used for long fragment amplification and the PCR reaction conditions were: 98 ℃ pre-denaturation for 2 min; 90–96 ℃ denaturation for 10 s; 50–62 ℃ annealing for 30 s; 60–72 ℃ extension for 10 min, 40 cycles, and 68 ℃ extension for 10 min. PCR products were purified using the Wizard SV Gel/PCR clean-up system (Promega) kit and sent to WinnerBio (Shanghai, China) for high-throughput sequencing using the Illumina Miseq PE250 platform with paired reads of 2 × 150 bp.

### Sequence assembly, annotation and analysis

The sequencing results were assembled using Geneious v.2020.2 Prime (created by Biomatters; available from https://www.geneious.com) according to the parameters: minimum overlap 50 bp, minimum overlap identity 98%. Finally, we obtained the complete mitochondrial genome of *E. huzhuensis* with an average coverage depth of 2371.04×. The assembled sequences were used to predict genes using Geneious Prime and the MITOS web server (Bernt et al. 2013). The 13 protein-coding genes were predicted using invertebrate mitochondrial genome codons in Geneious Prime, and then the 13 protein-coding genes were compared and edited using the BLAST tool provided by NCBI. ARWEN (Laslett and Canbäck 2008), tRNAscan-SE (Lowe and Eddy 1997), and the MITOS web server (Bernt et al. 2013) were used to identify 22 tRNA genes. Two rRNA genes were identified based on their putative secondary structures and previously sequenced mitochondrial genome sequences for comparison. The location of the control region was identified based on the boundaries of neighboring genes. The mitochondrial genome sequence of *E. huzhuensis* has been deposited in GenBank under accession number OQ067482.

The *E. huzhuensis* mitochondrial genome circular map was drawn using the website http://wolfe.ucd.ie/GenomeVx/. MEGA X was used to analyze the mitochondrial genome for nucleotide composition (Kumar et al. 2018). CodonW1.4.2 (https://sourceforge.net/projects/codonw/) determines the relative synonymous codon usage (RSCU) of 13 protein-coding genes.

### Phylogenetic analysis

*Limulus polyphemus* (JX983598) and *Carcinoscorpius rotundicauda* (MW446894) were selected as outgroups, and phylogenetic trees were constructed using MrBayes (Huelsenbeck and Ronquist 2001) and IQ-TREE (Nguyen et al. 2015) using the Bayesian inference (BI) method and the maximum likelihood (ML) method, respectively. Sequence comparison was performed using MAFFT (Katoh et al. 2002). Based on the Bayesian Information Criterion (BIC), ModelFinder2 (Kalyaanamoorthy et al. 2017) and PartitionFinder v.2.1.1 (Lanfear et al. 2017) were used to determine the best nucleotide models for constructing maximum likelihood (ML) and Bayesian trees, respectively (Tables S1 and S2). For the Bayesian systematics analysis, a total of 2,000,000 generations were run, sampling every 1,000 generations, burning the top 25% of the trees to ensure sample independence. Four Monte Carlo Markov Chains (MCMC) were also run. To estimate the support of the Bayesian trees, we calculated Bayesian posterior probabilities (PP). For maximum likelihood systematics analysis, we computed branch reliability (bootstrap probability, BP) using 50,000 ultra-fast bootstrap replications. The constructed phylogenetic tree was viewed and edited using FigTree v.1.4.4 (http://tree.bio.ed.ac.uk/software/figtree/). The species information used to construct the phylogenetic tree can be found in Table S3.

## Results and discussion

### Mitochondrial genome characterization of *Eulaelaps huzhuensis*

**Fig. 2:**
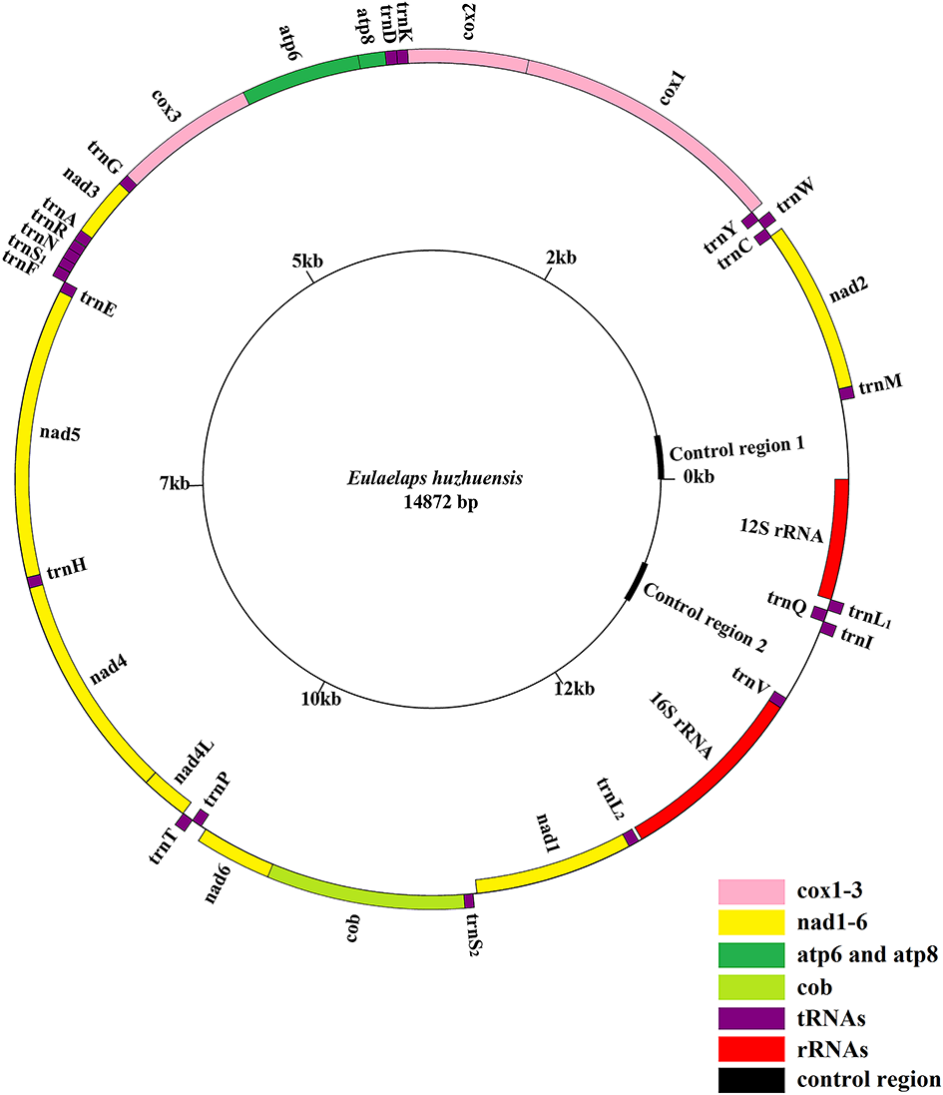
Circular map of *Eulaelaps huzhuensis* mitochondrial genome

The mitochondrial genome of *E. huzhuensis* is a double-stranded circular DNA molecule of 14,872 bp (Fig. 2). It is intermediate in size compared to the mitochondrial genomes of other Mesostigmata, which range from 14,423 bp (*Quadristernoseta* cf. *intermedia*) to 24,961 bp (*Metaseiulus occidentalis*) to date (Jeyaprakash and Hoy 2007; Li et al. 2019). It consists of 13 protein-coding genes (PCGs), 22 tRNA genes, two rRNA genes, and two control regions (Fig. 2). Mitochondrial genomes containing multiple control regions are common in some mites (e.g., *Coleolaelaps* cf. *liui*, *Hypoaspis linteyini*) and some insects (e.g., *Heterodoxus macropus*) (Shao et al. 2001; Li et al. 2019). Of these, 23 genes (nine PCGs and fourteen tRNAs) were encoded by the J strand, whereas the rest (four PCGs, eight tRNAs, and two rRNAs) were encoded by the N strand (Table 1). The base composition of *E. huzhuensis* was 34.2% for A, 33.8% for T, 20.2% for C and 11.8% for G, with an AT content of 68.0%, which was lower than the average A + T content of the Acari mitochondrial genome (75.3 ± 4.8%) (Yuan et al. 2010).

**Table 1:** Features of the mitochondrial genomes of *Eulaelaps huzhuensis*

*E. huzhuensis*	Position		Length (bp)	Coding strand	Nucleic acid		Codon		Anti codon	Amino acid size	Intergenic nucleotide
	Start	End			AT (%)	GC (%)	Start	Stop			
Mitogenome	1	14872	14872		68.0	32.0					
*CR1*	1	450	450		76.4	23.6					
*trnM*	451	515	65	+	63.1	36.9			CAU		
*nad2*	517	1473	957	+	68.1	31.9	ATT	TAA			1
*trnC*	1466	1526	61	-	80.3	19.7			GCA		-8
*trnW*	1527	1587	61	+	68.9	31.1			UCA		
*trnY*	1586	1646	61	-	70.5	29.5			GUA		-2
*cox1*	1648	3180	1533	+	63.1	36.9	ATT	TAA		511	1
*cox2*	3180	3851	672	+	65.5	34.5	ATG	TAA		224	-1
*trnK*	3852	3913	62	+	71.0	29.0			CUU		
*trnD*	3913	3976	64	+	79.7	20.3			GUC		-1
*atp8*	3977	4135	159	+	72.3	27.7	GTG	TAA		53	
*atp6*	4126	4791	666	+	64.9	35.1	ATG	TAA		222	-10
*cox3*	4792	5580	789	+	62.1	37.9	ATA	TAA		263	
*trnG*	5580	5641	62	+	88.7	11.3			UCC		-1
*nad3*	5642	5977	336	+	65.8	34.2	ATC	TAG		112	
*trnA*	5976	6036	61	+	77.0	23.0			UGC		-2
*trnR*	6041	6095	55	+	63.6	36.4			UCG		4
*trnN*	6094	6160	67	+	74.6	25.4			GUU		-2
*trnS*_*1*_	6153	6205	53	+	73.6	26.4			GCU		-8
*trnF*	6209	6269	61	+	80.3	19.7			GAA		3
*trnE*	6268	6328	61	-	77.0	23.0			UUC		-2
*nad5*	6329	8000	1672	-	68.4	31.6	ATA	T		557	
*trnH*	8001	8062	62	-	74.2	25.8			GUG		
*nad4*	8063	9365	1303	-	71.6	28.4	ATG	T		434	
*nad4L*	9367	9639	273	-	71.8	28.2	ATT	TAG		91	1
*trnT*	9641	9705	65	+	80.0	20.0			UGU		1
*trnP*	9706	9765	60	-	71.7	28.3			UGG		
*nad6*	9797	10231	435	+	67.4	32.6	ATC	TAA		145	31
*cob*	10228	11335	1108	+	61.6	38.4	ATA	T		369	-4
*trnS*_*2*_	11336	11388	53	+	66.0	34.0			UGA		
*nad1*	11410	12321	912	-	63.9	36.1	ATC	TAG		304	21
*trnL*_*2*_	12322	12382	61	-	60.7	39.3			UAA		
*16S rRNA*	12403	13498	1096	-	71.7	28.3					20
*trnV*	13499	13561	63	-	71.4	28.6			UAC		
*CR2*	13562	13983	422		76.3	23.7					
*trnI*	13984	14048	65	+	78.5	21.5			GAU		
*trnQ*	14046	14114	69	-	76.8	23.2			UUG		-3
*trnL*_*1*_	14116	14178	63	+	73.0	27.0			UAG		1
*12S rRNA*	14179	14872	694	-	71.8	28.2					

The *E. huzhuensis* mitochondrial genome showed a total of 10 intergenic regions (Table 1), and the longest intergenic sequence (31 bp) appeared between *trnP* and *nad6*, followed by *trnS*_*2*_ and *nad1* at 21 bp. It has been suggested that the intergenic region between *trnS*_*2*_ and *nad1* in the mitochondrial genome could serve as a transcription termination signal site in the mitochondrial genome during transcription (Cameron and Whiting 2008). So, it can be speculated that the transcription termination signal site of *E. huzhuensis* is located in the intergenic sequence adjacent to the *trnS*_*2*_ and *nad1* genes. In addition, 12 overlap regions were detected, with the longest overlap region (10 bp) located between *atp8* and *atp6* and the shortest overlap region (1 bp) occurring between six gene junctions. An overlap between *atp6* and *atp8* is very common in arthropod mitochondrial genomes (Ge et al. 2022). The presence of gene intergenic and overlap regions in the mitochondrial genome are presumed to beneficial for increasing the stability of the mitochondrial structure (Song et al. 2016).

### Protein-coding genes and codon usage

The total length of the 13 protein-coding genes was 10,815 bp, accounting for 72.7% of the complete mitochondrial genome. The length of the protein-coding genes ranged from 159 bp (*atp8*) to 1,672 bp (*nad5*), with AT/GC content ranging from 61.6/27.7% to 72.3/38.4% (Table 1). The 12 protein-coding genes have the typical ATN as the start codon (ATA:3, ATT:3, ATG:3, ATC:3), and only one protein-coding gene (*atp8*) shows a rare start codon (GTG). The rare start codon (GTG) has been reported in the mitochondrial genome of some species within the families Characidae, Agromyzidae, and Perlidae (Yang et al. 2013; Brandao-Dias et al. 2016; Cao et al. 2019). In the use of stop codons, 10 protein-coding genes ended with the complete stop codon TAA/TAG (TAA:7, TAG:3) and three protein-coding genes (*nad4*, *nad5*, *cob*) had the incomplete stop codon T. Incomplete stop codons are a common feature of many metazoa (Liu et al. 2019, 2022). These incomplete stop codons can be formed into complete stop codons TAA after transcription by polyadenylation (Huang et al. 2022).

**Fig. 3:**
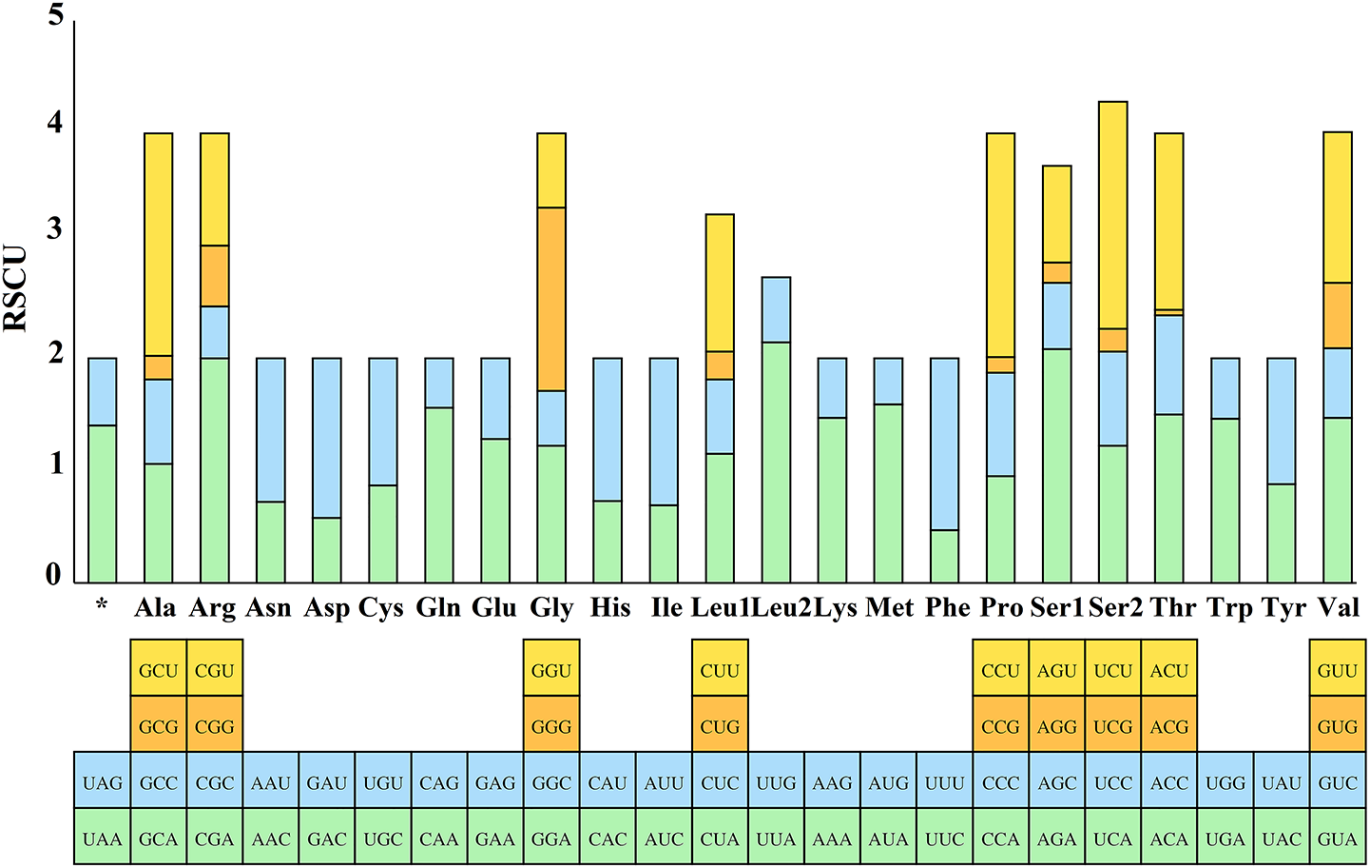
Relative synonymous codon usage (RSCU) of *Eulaelaps huzhuensis*. The Y-axis indicates the RSCU value, and the X-axis indicates the codons corresponding to the respective amino acids

Relative synonymous codon usage (RSCU) was calculated for the *E. huzhuensis* mitochondrial genome (Fig. 3). RSCU values indicate strong codon usage preference (RSCU > 1), no preference (RSCU = 1), and weak preference (RSCU < 1; Liu et al. 2016). The most commonly used codons are UUA (Leu) and AGA (Ser), whereas ACG (Thr) is the least commonly used codon. Most codons ending in A/U have RSCU > 1, whereas codons ending in G/C have RSCU < 1, which is similar to that in other metazoa (Hao et al. 2017; Wu et al. 2022).

### Transfer RNA, ribosomal RNA genes and control region

**Fig. 4:**
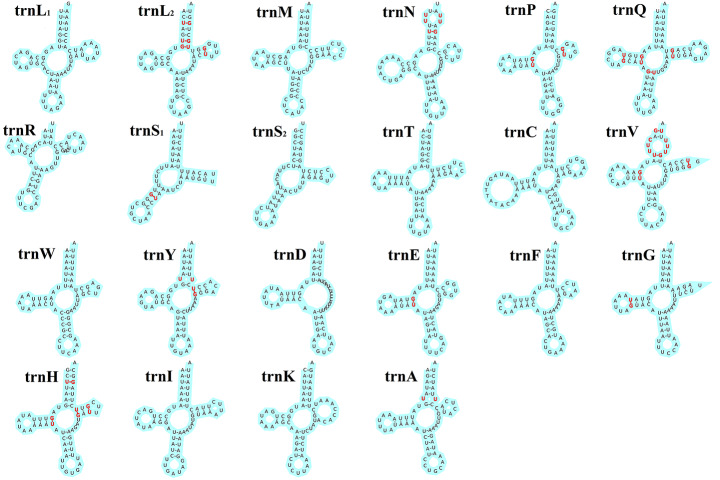
Putative secondary structure of 22 tRNA genes in *Eulaelaps huzhuensis*. Bold red indicates mismatch

The size of *E. huzhuensis* 22 tRNA genes ranged from 53 bp (*trnS*_*1*_ and *trnS*_*2*_) to 69 bp (*trnQ*), and their secondary structures are shown in Fig. 4. Three tRNA genes had missing arms, i.e., *trnS*_*1*_ and *trnS*_*2*_ were missing the D arm and *trnD* was missing the T arm, whereas the remaining 19 tRNA genes are all capable of forming typical clover-leaf secondary structures. Some scholars have suggested that tRNA gene secondary structure changes may be linked to the evolution of species (Yokogawa et al. 1991; Watanabe et al. 1994). However, the validity of this claim needs further in-depth study. The anticodon of most arthropod *trnS*_*1*_ is GCU, whereas *E. huzhuensis* uses UCU as the anticodon of *trnS*_*1*_. This exceptional anticodon of *trnS*_*1*_ is also found in many other beetles (Du et al. 2017). During tRNA gene folding, in addition to the typical Waston-Crick pairing (A-U, G-C), there are 30 mismatched base pairs, of which G-U pairing occurs up to 23×. G-U mismatches most often occur at the junction of the single-stranded V-loop and T-helix in tRNA genes, because there the polynucleotide strand makes a sharp turn and G-U mismatches can stabilize the rotation of the backbone (Clark and Klug 1975). Of the remaining mismatches, U-U mismatches occurred 6×, and 1× it was a U-C mismatch. Mismatches in tRNA genes are common, and these mismatched bases can be corrected by post-transcriptional editing and do not affect the transport function of tRNA genes (Reichert and Mörl 2000).

The two rRNA genes (*16S rRNA* and *12S rRNA*) were 1,096 and 694 bp in length, respectively (Table 1), similar to other mites (Li et al. 2019). The *16S rRNA* gene was located between *trnL*_*2*_ and *trnV* with AT/GC content of 71.7/28.3%; the *12S rRNA* gene was located between *trnL*_*1*_ and CR1 with AT/GC content of 71.8/28.2%.

Duplicated control regions (CR1 and CR2) were identified in the *E. huzhuensis* mitochondrial genome. CR1 and CR2 are 450 and 422 bp in length, respectively, and share a common core sequence of 422 bp (CR1 at positions 18–439 and CR2 at positions 13,562–13,983). CR1 is located between *12S rRNA* and *trnM* with AT/GC content of 76.4/23.6%, respectively; CR2 is located between *trnV* and *trnI* with AT/GC content of 76.3/23.7%, respectively.

### Gene rearrangement

**Fig. 5:**
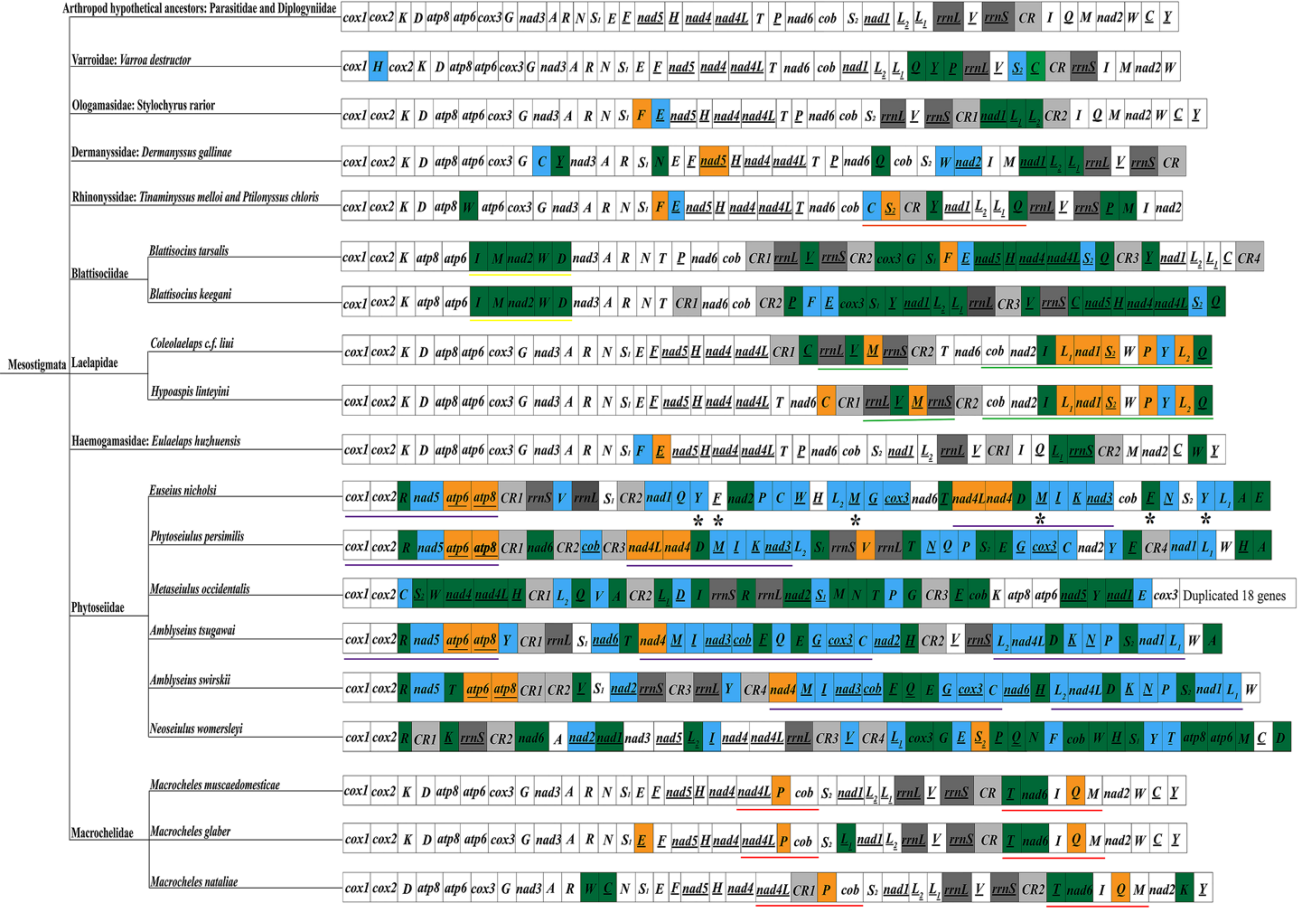
Mitochondrial genome arrangement pattern of Mesostigmata. Black underline indicates that the gene is located on the N-strand; blue indicates that the gene is inverted and translocated; green indicates that the gene is translocated; orange indicates that the gene is inverted. Two rRNA genes are indicated in dark gray; control region is indicated in light gray. ✱ indicates a duplicated gene; same color underlines indicate shared gene clusters

The phenomenon of mitochondrial genome rearrangements is generally considered an important tool to resolve phylogenetically unstable populations (Boore et al. 1995, 1998). Gene rearrangements are more common in invertebrate mitochondrial genomes, such as those of insects and mites (Babbucci et al. 2014; Li et al. 2019; Zhang et al. 2021). Using the mitochondrial genome arrangement of the hypothetical arthropod ancestor (*L. polyphemus*) as a reference (Staton et al. 1997), the gene arrangement order in Mesostigmata was analyzed (Fig. 5). It was found that only species in the families Parasitidae and Diplogyniidae had the same arrangement of mitochondrial genomes as the hypothetical arthropod ancestor. However, the remaining nine families (Varroidae, Ologamasidae, Dermanyssidae, Laelapidae, Haemogamasidae, Blattisociidae, Rhinonyssidae, Macrochelidae and Phytoseiidae) with 19 species showed different degrees of rearrangements, and the highest rate of rearrangements occurred in the family Phytoseiidae. More importantly, it was found that some species of the same family or genus share the same gene clusters, such as the family Laelapidae sharing two gene clusters, i.e., *rrnL*-*trnV*-*trnM-rrnS* and *cob*-*nad2*-*trnI*-*trnL*_*1*_-*nad1*-*trnS*_*2*_-*trnW*-*trnP*-*trnY*-*trnL2*-*trnQ* (underlining indicates that the gene is located on a different strand); species in the family Rhinonyssidae share one gene cluster, which is *trnC*-*trnS*_*2*_-*trnY*-*nad1*-*trnL*_*2*_-*trnL*_*1*_-*trnQ*; species of the genus *Blattisocius* (Blattisociidae) share one gene cluster, namely *trnI*-*trnM*-*nad2*-*trnW*-*trnD*; three species of the genus *Macrocheles* (Macrochelidae) also share two gene clusters, that is, *nad4L*-*trnP*-*cob* and *trnT*-*nad6*-*trnI*-*trnQ-trnM*; in addition, three species (*Phytoseiulus persimilis*, *Euseius nicholsi* and *Amblyseius tsugawai*) of the family Phytoseiidae share one gene cluster, which is *cox1*-*cox2*-*trnR*-*nad5*-*atp6*-*atp8*, *P. persimilis* and *E. nicholsi* also share one gene cluster, i.e., *nad4L*-*nad4*-*trnD*-*trnM*-*trnI*-*trnK*-*nad3*, and two additional gene clusters are shared by species of the genus *Amblyseius*, namely *nad4*-*trnM*-*trnI*-*nad3*-*cob*-*trnF*-*trnQ*-*trnE*-*trnG*-*cox3*-*trnC* and *trnL*_*2*_-*nad4L*-*trnD*-*trnK*-*trnN*-*trnP*-*trnS*_*2*_-*nad1*-*trnL*_*1*_.

The above results suggest that these same gene clusters are common derivatives of these families or genera, and differences exist between gene clusters of different families or genera. In this study, *E. huzhuensis* had five genes rearranged in its mitochondrial genome. This is a new rearrangement pattern in Mesostigmata and does not share gene clusters with other families. Rearrangement in species of the family Varroidae is associated only with tRNA genes, whereas rearrangement in other species is also associated with protein-coding genes. It has been confirmed that insect mitochondrial genomes have a much higher frequency of tRNA gene rearrangements than large protein-gene clusters (Cameron 2014). Moreover, many rearrangements occur near the control region, which contains regulatory elements essential for replication and transcription, resulting in the vicinity of the control region being a hotspot for rearrangements. From these results, it can be inferred that these rearranged genes or regions may become hotspots for the study of Mesostigmata species mitochondrial genomes.

### Phylogenetic analysis

**Fig. 6:**
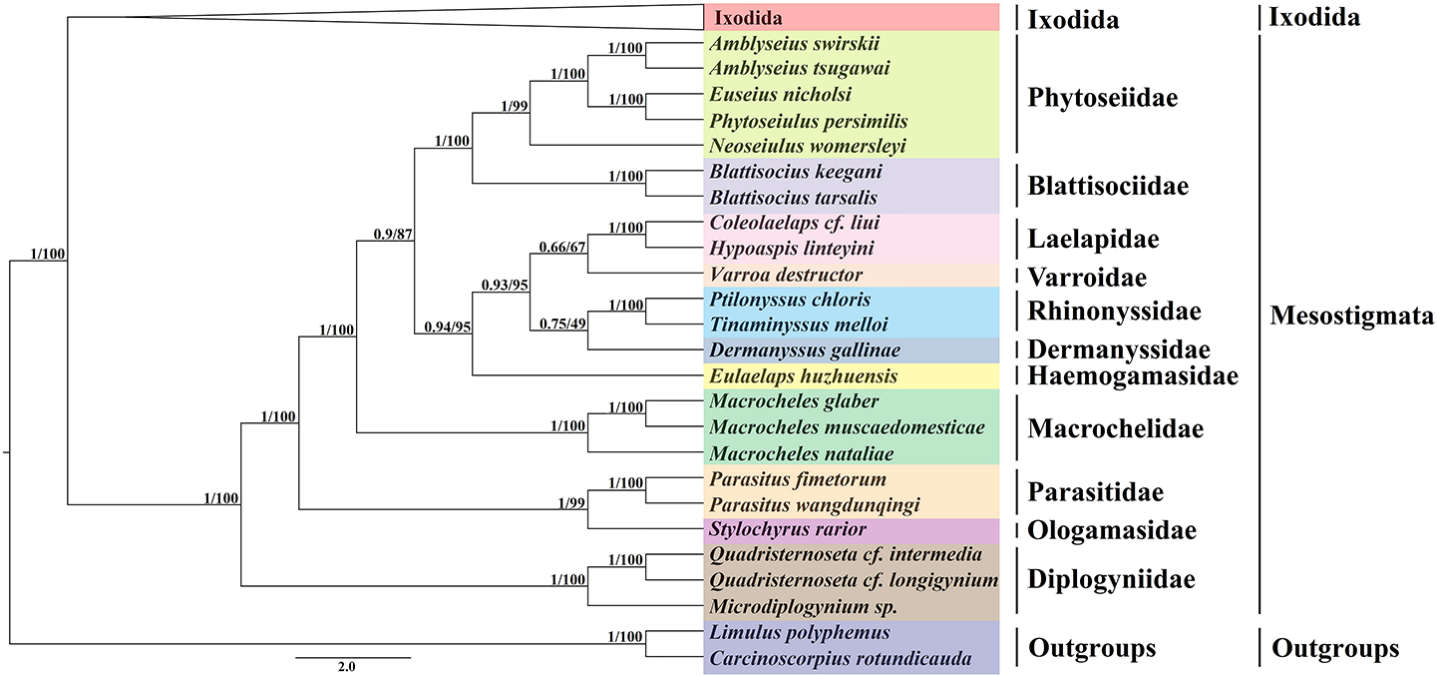
Phylogenetic tree constructed based on 13 mitochondrial protein-coding genes. The numbers next to the nodes represent Bayesian inference probabilities (BI) and maximum bootstrap (ML), respectively

The short length of single genes contains limited phylogenetic information, and single genes have different abilities to reconstruct phylogenies in different biological groups, leading to difficulties in a true reflection of the phylogenetic relationships in species (Cheng et al. 2013; Mirarab 2017; Jiang et al. 2021). Thus, based on complete mitochondrial genomic data for phylogenetic analysis it is more comprehensive to reflect the molecular evolutionary level of species (Liu et al. 2019; Wang et al. 2019, 2020). In this study, a phylogenetic tree was constructed based on 13 protein-coding genes using maximum likelihood and Bayesian methods with *L. polyphemus* and *C. rotundicauda* (Limulidae) as outgroups (Fig. 6). The phylogenetic trees constructed by the two analytical methods formed identical topologies and formed nodes with high support rates. In summary, the maximum likelihood tree (ML) was less supportive than the Bayesian tree (BI). Both the BI and ML trees support that Mesostigmata is a monophyletic group (BP = 100, PP = 1), consistent with the findings of Li et al. (2019). It overturned the conclusion of Lindquist et al. (2009b) that Mesostigmata is a polyphyletic group. The phylogenetic relationships among all families are clear, and the three species of the family Diplogyniidae are located at the base of the phylogenetic tree, indicating that the family Diplogyniidae is an early divergent taxon in Mesostigmata. In the present study, *E. huzhuensis* did not cluster with species of the family Laelapidae, but formed a monophyletic branch, further suggesting from molecular data that the family Haemogamasidae is a separate family and not a subfamily of the family Laelapidae.

## Conclusion

The *E. huzhuensis* mitochondrial genome is a circular double-stranded molecule containing 37 genes and two control regions. It needs to be deeply explored whether the inability of some tRNA genes to form a typical secondary structure is related to the evolution of the species. Besides, the rearrangement of the *E. huzhuensis* mitochondrial genome is a new type of rearrangement in Mesostigmata. Whether the arrangement pattern of *E. huzhuensis* mitochondrial genomes is a common derivation of the genus *Eulaelaps* or the family Haemogamasidae remains to be verified by sequencing mitochondrial genomes of more species of the family Haemogamasidae. The phylogenetic analysis constructed based on protein-coding gene data strongly supports the monophyly of the family Haemogamasidae. Our results provide new insights into the molecular evolution of the family Haemogamasidae, as well as useful information for gaining insight into the mechanism of Mesostigmata rearrangement.

## Electronic Supplementary Material

Below is the link to the electronic supplementary material.

Supplementary Material 1

Supplementary Material 2

Supplementary Material 3

## Data Availability

The datasets generated during the current study are available in the National Center for Biotechnology Information at https://www.ncbi.nlm.nih.gov. Accession numbers is: OQ067482.
