# Supplementary material for: The complete mitochondrial genome of Eulaelaps huzhuensis (Mesostigmata: Haemogamasidae)
Source: Exp Appl Acarol. 2023 Jun 22;90(3-4):301–16. doi: 10.1007/s10493-023-00802-6 (PMC10406673; doi:10.1007/s10493-023-00802-6)
Supplement: Supplementary file 1 — Supplementary Material 1 [file 10493_2023_802_MOESM1_ESM.docx]

**Supplementary material**

**Table S1.** The best partitioning scheme and best-fit model for constructing an IQ-TREE were obtained using ModelFinder2.

| **Best Model** | **Genes and codon positions by ModelFinder2** |
| --- | --- |
| GTR+F+I+I+R5 | atp6_codon_pos1, nad3_codon_pos1 |
| TVM+F+R5 | atp6_codon_pos2, cox2_codon_pos2, cox3_codon_pos2, cytb_codon_pos2, nad3_codon_pos2 |
| TIM+F+R5 | atp6_codon_pos3, NAD2_codon_pos3, nad3_codon_pos3, nad6_codon_pos3 |
| TN+F+I+I+R3 | atp8_codon_pos1 |
| TIM2+F+I+I+R4 | atp8_codon_pos2, atp8_codon_pos3 |
| GTR+F+I+I+R4 | cox1_codon_pos1 |
| GTR+F+I+G4 | cox1_codon_pos2 |
| HKY+F+R6 | cox1_codon_pos3, cox2_codon_pos3, cox3_codon_pos3 |
| GTR+F+I+I+R5 | cox2_codon_pos1, cox3_codon_pos1, cytb_codon_pos1 |
| TIM2+F+ASC+G4 | cytb_codon_pos3 |
| TIM2+F+I+I+R5 | nad1_codon_pos1, nad4L_codon_pos1, nad4_codon_pos1, nad5_codon_pos1 |
| GTR+F+I+I+R4 | nad1_codon_pos2, nad4L_codon_pos2, nad4_codon_pos2, nad5_codon_pos2 |
| TPM2u+F+ASC+R5 | nad1_codon_pos3 |
| TIM+F+R5 | nad_codon_pos1, nad6_codon_pos1 |
| GTR+F+I+G4 | nad2_codon_pos2, nad6_codon_pos2 |
| K3Pu+F+ASC+R4 | nad4L_codon_pos3 |
| TPM3u+F+ASC+R5 | nad4_codon_pos3, nad5_codon_pos3 |
