# Supplementary material for: The complete mitochondrial genome of Eulaelaps huzhuensis (Mesostigmata: Haemogamasidae)
Source: Exp Appl Acarol. 2023 Jun 22;90(3-4):301–16. doi: 10.1007/s10493-023-00802-6 (PMC10406673; doi:10.1007/s10493-023-00802-6)
Supplement: Supplementary file 2 — Supplementary Material 2 [file 10493_2023_802_MOESM2_ESM.docx]

**Table S2.** The best partitioning scheme and best-fit model for constructing a BI were obtained using PartitionFinder2.1.1.

| **Best Model** | **Genes and codon positions by PartitionFinder2.1.1** |
| --- | --- |
| GTR+I+G | nad1_codon_pos1, nad4_codon_pos1, nad4L_codon_pos1, nad5_codon_pos1 |
| GTR+I+G | nad4L_codon_pos2, nad4_codon_pos2, nad1_codon_pos2, nad5_codon_pos2 |
| HKY+G | nad1_codon_pos3 |
| GTR+I+G | nad6_codon_pos1, atp8_codon_pos1, nad2_codon_pos1 |
| GTR+I+G | atp8_codon_pos2, nad6_codon_pos2, nad2_codon_pos2 |
| HKY+G | nad6_codon_pos3, nad2_codon_pos3 |
| GTR+I+G | atp6_codon_pos1, nad3_codon_pos1, cytb_codon_pos1, cox3_codon_pos1, cox2_codon_pos1 |
| GTR+I+G | cytb_codon_pos2, cox2_codon_pos2, cox3_codon_pos2, atp6_codon_pos2, nad3_codon_pos2 |
| HKY+G | nad3_codon_pos3, atp6_codon_pos3, atp8_codon_pos3 |
| GTR+G | nad4_codon_pos3, nad4L_codon_pos3, nad5_codon_pos3 |
| GTR+I+G | cox1_codon_pos1 |
| GTR+I+G | cox1_codon_pos2 |
| GTR+G | cox2_codon_pos3, cox1_codon_pos3, cox3_codon_pos3 |
| HKY+I+G | cytb_codon_pos3 |
