# Supplementary material for: The complete mitochondrial genome of Eulaelaps huzhuensis (Mesostigmata: Haemogamasidae)
Source: Exp Appl Acarol. 2023 Jun 22;90(3-4):301–16. doi: 10.1007/s10493-023-00802-6 (PMC10406673; doi:10.1007/s10493-023-00802-6)
Supplement: Supplementary file 3 — Supplementary Material 3 [file 10493_2023_802_MOESM3_ESM.docx]

**Table S3**. Species information for constructing phylogenetic trees.

| Family | Genus | Species | GenBank no. |
| --- | --- | --- | --- |
| Ixodidae | *Dermacentor* | *Dermacentor nuttalli* | NC028528 |
|  |  | *Dermacentor silvarum* | NC026552 |
|  |  | *Dermacentor marginatus* | MK905212 |
|  |  | *Dermacentor everestianus* | NC042764 |
|  |  | *Dermacentor reticulatus* | MT478096 |
|  |  | *Dermacentor auratus* | NC059724 |
|  | *Rhipicephalus* | *Rhipicephalus australis* | NC023348 |
|  |  | *Rhipicephalus microplus* | NC023335 |
|  |  | *Rhipicephalus geigyi* | NC023350 |
|  |  | *Rhipicephalus camicasi* | NC061616 |
|  |  | *Rhipicephalus simus* | KY457542 |
|  | *Hyalomma* | *Hyalomma marginatum* | MW366632 |
|  |  | *Hyalomma rufipes* | KY457528 |
|  |  | *Hyalomma truncatum* | KY457529 |
|  |  | *Hyalomma asiaticum asiaticum* | MF101817 |
|  | *Amblyomma* | *Amblyomma javanense* | NC043872 |
|  |  | *Amblyomma testudinarium* | MT029329 |
|  |  | *Aponomma fimbriatum* | NC017759 |
|  |  | *Amblyomma geoemydae* | MK814531 |
|  |  | *Amblyomma maculatum* | MW719251 |
|  | *Haemaphysalis* | *Haemaphysalis longicornis* | NC037493 |
|  |  | *Haemaphysalis bancrofti* | NC041076 |
|  |  | *Haemaphysalis concinna* | NC034785 |
|  | *Archaeocroton* | *Archaeocroton sphenodonti* | NC017745 |
|  | *Bothriocroton* | *Bothriocroton concolor* | NC017756 |
|  |  | *Bothriocroton undatum* | NC017757 |
|  | *Robertsicus* | *Robertsicus elaphensis* | NC017758 |
|  | *Ixodes* | *Ixodes cornuatus* | NC062630 |
|  |  | *Ixodes myrmecobii* | NC062632 |
|  |  | *Ixodes holocyclus* | NC005293 |
|  |  | *Ixodes hirsti* | NC062631 |
|  |  | *Ixodes trichosuri* | NC062633 |
|  |  | *Ixodes uriae* | NC006078 |
|  |  | *Ixodes fecialis* | NC062628 |
| Nuttalliellidae | *Nuttalliella* | *Nuttalliella namaqua* | JQ665719 |
| Phytoseiidae | *Amblyseius* | *Amblyseius swirskii* | MW729377 |
|  |  | *Amblyseius tsugawai* | MW729376 |
|  | *Euseius* | *Euseius nicholsi* | KM999989 |
|  | *Phytoseiulus* | *Phytoseiulus persimilis* | GQ222414 |
|  | *Neoseiulus* | *Neoseiulus womersleyi* | MW762685 |
| *Blattisociidae* | *Blattisocius* | *Blattisocius keegani* | MH120211 |
|  |  | *Blattisocius tarsalis* | MK270529 |
| Laelapidae | *Coleolaelaps* | *Coleolaelaps* cf. *liui* | MK270524 |
|  | *Hypoaspis* | *Hypoaspis linteyini* | MK270530 |
| Varroidae | *Varroa* | *Varroa destructor* | AJ493124 |
| Rhinonyssidae | *Ptilonyssus* | *Ptilonyssus chloris* | MN557819 |
|  | *Tinaminyssus* | *Tinaminyssus melloi* | MN557820 |
| Dermanyssidae | *Dermanyssus* | *Dermanyssus gallinae* | MW044618 |
| Haemogamasidae | *Eulaelaps* | *Eulaelaps huzhuensis* | OQ067482 |
| Macrochelidae | *Macrocheles* | *Macrocheles glaber* | MK270525 |
|  |  | *Macrocheles muscaedomesticae* | MK270526 |
|  |  | *Macrocheles nataliae* | MK270527 |
| Parasitidae | *Parasitus* | *Parasitus fimetorum* | OK572962 |
|  |  | *Parasitus wangdunqingi* | MK270528 |
| Ologamasidae | *Stylochyrus* | *Stylochyrus rarior* | GQ927176 |
| Diplogyniidae | *Quadristernoseta* | *Quadristernoseta* cf*. intermedia* | MK270521 |
|  |  | *Quadristernoseta* cf*. longigynium* | MK270522 |
|  |  | *Microdiplogynium* sp*.* | MK270523 |
